# Generation of iPSC and isogenic gene-corrected lines from a patient with Shwachman Diamond syndrome

**DOI:** 10.1016/j.scr.2026.104045

**Published:** 2026-06-25

**Authors:** Parisa Ghiasighorveh, Summer Moore, Alyssa L. Kennedy

**Affiliations:** a Department of Pathology, St. Jude Children’s Research Hospital, Memphis, TN, USA; b Department of Hematology, St. Jude Children’s Research Hospital, Memphis, TN, USA

## Abstract

Shwachman Diamond Syndrome (SDS) is an inherited bone marrow failure and leukemia predisposition syndrome characterized by exocrine pancreatic insufficiency, skeletal dysplasia and bone marrow failure. SDS is inherited in an autosomal recessive manner and most patients with SDS carry biallelic mutations in the *SBDS* gene. We generated an iPSC line from a patient biallelic *SBDS* mutations (*c.258* + 2 T > C & c.183–184delinsCT(p.Lys62*) along with a corresponding isogenic control line correcting the splice site mutation with a cytosine base editor.

## Resource Table

**Table:** 

**Unique stem cell line identifiers**	STJUDEi009-A, STJUDEi009-A-1
**Alternative name(s) of stem cell lines**	SBDS1, SBDS1-iso
**Institution**	St. Jude Children’s Research Hospital
**Contact information of the reported cell line distributor**	Alyssa L. Kennedy
**Type of cell lines**	iPSCs
**Origin**	human
**Additional origin info (*applicable for human ESC or iPSC*)**	Age: 11 year oldSex: female
**Cell Source**	Bone marrow stromal fibroblasts
**Method of reprogramming (if applicable)**	Transient expression of mRNAs: OCT4, SOX2, KLF4, c-MYC, NANOG, LIN28
**Evidence of the reprogramming transgene loss (including genomic copy absence/removal/silencing, if applicable) (if applicable)**	N/A
**The cell culture system used**	Manual/EDTA based aggregate passaging. Colonies were not grown on MEFs nor did we culture with or use MEF-conditioned medium. Cells are maintained in StemFlex for Feeder-Free PSC Culture
**Genetic Features: Introduced and Pre-Existing**	Pre-existing mutation in *SBDS* c.258 + 2 T > C & c.183-184delinsCT (p.Lys62*)
**Associated disease**	Shwachman Diamond Syndrome – OMIM code #260400
**Gene/locus modified in the reported transgenic lines**	SBDS1 – *SBDS* c.258 + 2 T > CSDBS1 iso – edited to wild type (*SBDS* c258 + 2 T)
**Method of modification / user-customizable nucleases (UCN) used, *in silico* resources used for design optimization**	Cytosine base editing using CBE6b as described by Zhang et al.^1^ The construct was further modified for our application by replacing the canonical SpCas9 with SpRY Cas9 to enable PAM-flexible targeting, as no suitable NGG PAM site was available near the c.258 + 2 locus.In silico design involved manual sequence inspection and alignment to the reference locus to ensure optimal placement within the editing window. Potential off-target sites were evaluated using CRISPOR and Cas-OFFinder.
**User-customizable nuclease (UCN) delivery method**	mRNA delivered via electroporation
**All double-stranded DNA genetic material molecules are administered to the cells**	N/A
**Evidence of the absence of random integration of any plasmids or dsDNA introduced into the cells**	N/A
**Analysis of the resulting nuclease-targeted allele status**	Nested PCR of the SBDS gene followed by Sanger sequencing and NGS.
**Homozygous allele status validation**	PCR spanning the editing site followed by Sanger and NGS sequencing.
**Method of the off-target nuclease activity prediction and surveillance**	Guide RNA (gRNA) sequences were manually designed and evaluated using CRISPOR and Cas-OFFinder to identify potential off-target binding sites across the genome. Predicted sites were ranked based on CFD scores, mismatch number, and genomic context.On-target editing outcomes were assessed by targeted deep sequencing (NGS) following partial amplification of the SBDS locus. These analyses demonstrated efficient on-target editing with no detectable bystander edits or insertions/deletions (indels) within the sequenced region.Predicted off-target sites were analyzed separately by targeted PCR amplification followed by targeted deep sequencing of selected top-ranked loci.
**Descriptive name of the transgene**	N/A
**Eukaryotic selective agent resistance cassettes (including inducible, gene/cell type-specific)**	N/A
**Inducible/constitutive expression system details**	N/A
**Date archived/stock creation date**	Master banks were created November 2025.
**Cell line repository/bank**	STJUDEi009-Ahttps://hpscreg.eu/cell-line/STJUDEi009-ASTJUDEi009-A-1https://hpscreg.eu/cell-line/STJUDEi009-A-1
**Ethics/GMO work approvals**	The St. Jude institutional review board approved the biobanking of patient cells and generation of iPSCs under IRB #Pro00006262 “INSIGHT-HD: Investigation of the genetics of hematological diseases.”
**Disclaimers for Addgene/repository-sourced used recombinant DNA resources (if applicable)**	N/A

## 1. Resource utility

Shwachman Diamond Syndrome is an inherited bone marrow failure and leukemia predisposition syndrome caused by biallelic mutations in the SBDS gene. iPSC models are needed to understand the etiology of bone marrow failure in this disorder and to develop novel therapies with the correct genomic context found in patients.

## 2. Resource details

Shwachman-Diamond syndrome (SDS) is an inherited ribosomopathy characterized by bone marrow failure, exocrine pancreatic insufficiency, and skeletal abnormalities (Shwachman et al., 1964; Bodian et al., 1964). Most cases of SDS are caused by biallelic mutations in the *SBDS* gene and are inherited in an autosomal recessive manner (Boocock, 2003). The most common mutation, *SBDS* c.258 + 2T > C, disrupts the canonical 5′ splice site at the exon 2–intron 2 junction, resulting in aberrant splicing. This leads to exon skipping or use of a cryptic splice site upstream, producing truncated transcripts and frameshift mutations. Patients with SDS are at high risk for progression to myelodysplastic syndrome (MDS) and acute myeloid leukemia (AML), with biallelic *TP53* mutations now recognized as key drivers of malignant transformation (Kennedy, 2021). While pancreatic insufficiency can be managed with enzyme replacement, hematologic complications are more challenging; hematopoietic stem cell transplantation remains the only curative therapy (Myers, 2020). Bone marrow samples from patients with SDS show a marked reduction in hematopoietic stem and progenitor cells, limiting experimental study. Patient-derived induced pluripotent stem cell (iPSC) models provide a critical platform to study disease mechanisms in the appropriate human genetic context and to develop novel therapeutic approaches.

We generated SBDS-1 *(*STJUDEi009-A), an iPSC line from a pediatric patient with SDS and cytopenias. This patient had inherited biallelic pathogenic SBDS mutations (*SBDS* c.258 + 2 T > C & c.183–184delinsCT(p.Lys62*)). Bone marrow stromal fibroblasts taken from a surveillance marrow biopsy were cultured to obtain primary tissue. iPSC lines were generated by transient, sequential mRNA transfection of patient-derived bone marrow stromal cells with reprogramming factors OCT3/4, SOX2, KLF4, c-MYC, NANOG, LIN28. iPSC colonies were manually selected based on compact morphology, well-defined round edges, and high nucleus-to-cytoplasm ratios. These colonies were expanded in the presence of ROCK inhibitor and subsequently genotyped and karyotyped. Using a cytosine base editor (Zhang et al., 2024) sgRNA combination, we next corrected the *SBDS* c.258 + 2 T > C transition mutation to create SBDS1-iso. To confirm restoration of the wild type allele in edited iPSC clones, we performed NGS and Sanger sequencing (Fig. 1A). PCR followed by next-generation sequencing showed no indels/deletions or bystander edits in the editing window. Off-target analysis was performed using deep targeted NGS of the top five predicted off-target sites. The highest-ranked site showed ~4% off-target editing, while no detectable off-target activity was observed at the remaining four sites. These results indicate minimal off-target effects overall (Supplemental Fig. 1B) Each line demonstrates typical iPSC morphology and high post-thaw viability (Fig. 1B). In addition, pluripotency markers were evaluated via flow cytometry for stemness markers including SOX2, OCT3/4, NANOG, TRA-1–60, and SSEA-3 (Fig. 1C) (Tables 1 and 2). Each line also demonstrates trilineage in vitro differentiation capacity as assessed by ectoderm marker PAX6, endoderm marker FOXA2, and mesoderm marker Brachyury (Fig. 1D). Karyotyping of edited and unedited clones was normal, and both lines were negative for mycoplasma (Fig. 1E & Supplementary Fig. 1A). These studies establish an isogenic pair of patient-derived SDS iPSC that can be used for further mechanistic and translational studies.

## 3. Materials and Methods

### 3.1. Reprogramming, iPSC Culture, and trilineage differentiation

Bone marrow stromal cells were isolated from syringe washes obtained during bone marrow aspirate procedures, minimally expanded (passages 3–4), and reprogrammed using the StemRNA 3rd Gen Reprogramming Kit (ReproCELL) following manufacturer instructions. Individual iPSC colonies were selected and maintained under feeder-free conditions on Geltrex-coated plates (1:150 in DMEM/F12) in StemFlex Medium at 37°C and 5% CO_2_. Cells were passaged every 4 days using ReLeSR, and routine mycoplasma testing was performed using the ATCC Universal Mycoplasma Detection Kit (Passage 22 and every 4 passages). Pluripotency and differentiation potential were assessed using the STEMdiff Trilineage Differentiation Kit, with lineage specification confirmed by immunofluorescence staining for Brachyury (mesoderm), FOXA2 (endoderm), and PAX6 (ectoderm).

### 3.2. Gene editing and sequencing

SBDS-mutant iPSCs were dissociated using TrypLE Express and nucleofected (Lonza 4D-Nucleofector) with mRNA and sgRNA (250 ng/μL each) using P3 solution and program CB-150. Approximately 1 × 10^5^ cells were transfected per reaction. Four days after nucleofection, cells were plated at low density on Geltrex-coated plates for colony formation and cultured in StemFlex medium supplemented with CloneR2 followed by complete medium. Individual colonies were manually picked, expanded, and analyzed by targeted deep sequencing on an Illumina MiSeq platform. Sequence data were analyzed using CRIS.py (Connelly and Pruett-Miller, 2019), and correctly edited clones were confirmed by Sanger sequencing (Eurofins Genomics). Genomic DNA was isolated using the PureLink Genomic DNA Mini Kit, PCR-amplified (438 bp product), and chromatograms analyzed using SnapGene.

### 3.3. Karyotyping and STR analysis

Chromosomal integrity was assessed by standard cytogenetics following nocodazole treatment, with at least 40 metaphases analyzed per line. Short tandem repeat (STR) profiling using the PowerPlex Fusion System (Promega) confirmed sample identity across 24 loci. Karyotyping was performed at passage 15 (SBDS1) and passage 3 (SBDS1-iso).

### 3.4. Immunofluorescence

Passage 17 (SBDS1) and passage 5 (SBDS1-iso) cells were plated on Geltrex-coated chamber slides and, following trilineage differentiation, fixed and permeabilized using BD Cytofix/Cytoperm. After blocking with 3% BSA, cells were incubated with primary and secondary antibodies, counterstained with one drop per chamber of Prolong Gold Antifade with DAPI, and imaged using an ECHO Revolve microscope.

### 3.5. Flow cytometry

Passage 20 (SBDS1) and passage 5 (SBDS1-iso) iPSC colonies were dissociated with TrypLE Express and analyzed for pluripotency markers by flow cytometry. Surface markers included TRA-1–60 and SSEA-3; intracellular markers included NANOG, SOX2, and OCT3/4 following fixation and permeabilization with BD Cytofix/Cytoperm. Aquired on LSR Fortesa instrument and analysed with FlowJo version 10 software.

## Supplementary Material

Supplementary Material

## Figures and Tables

**Fig. 1.**
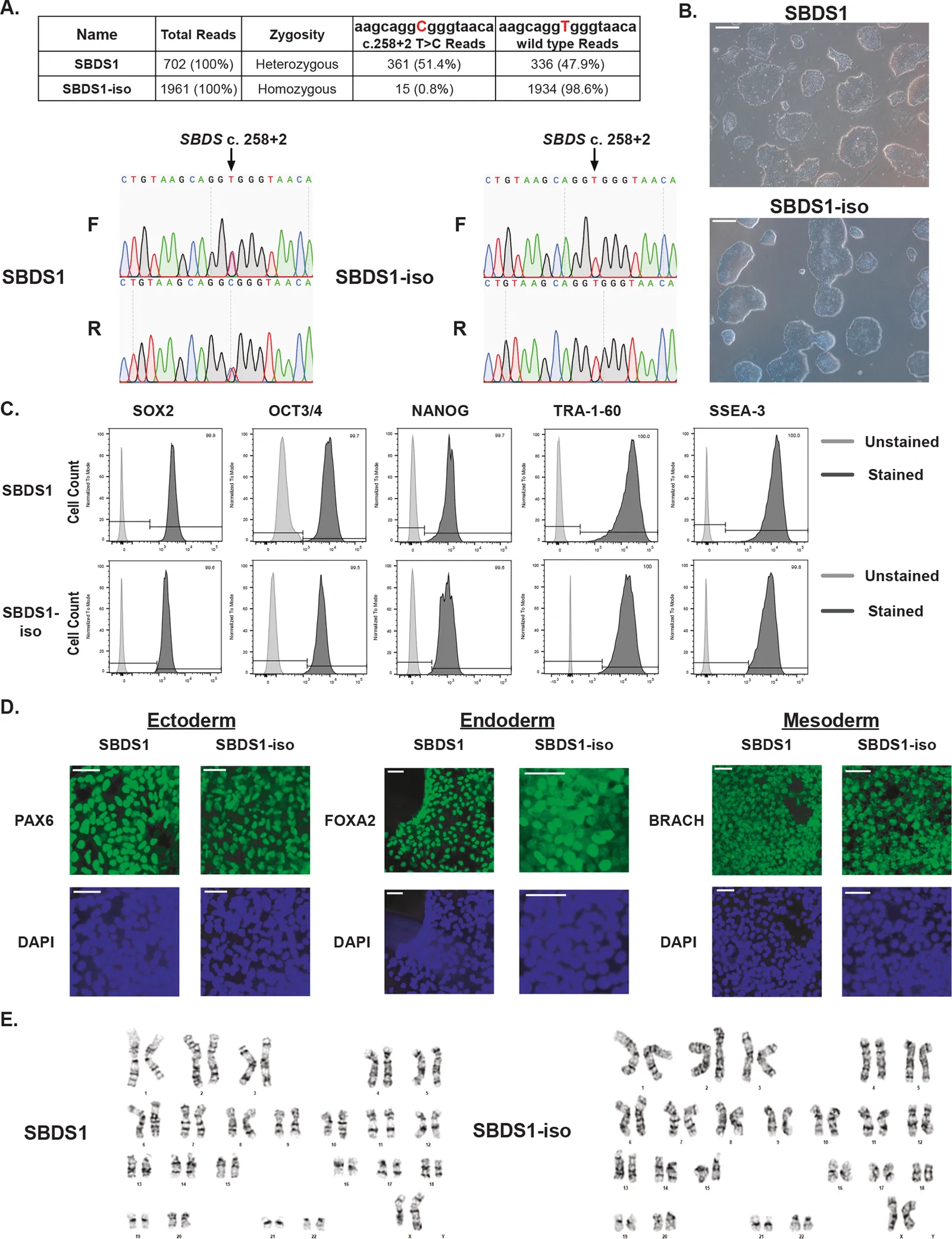
Characterization of SDS patient-derived iPSCs (SBDS c.258 + 2 T > C) and isogenic corrected line.

**Table 1:** Characterisation and validation.

Classification	Output type	Result	Data (suggested placement)

**Schematic of a transgene/genetic modification**	Schematic illustrating the structure and location of the introduced genetic modification	SBDS-1 is heterozygous for the c.258 + 2 T > C mutation, whereas SBDS-1 iso is homozygous for the wild-type c.258 + 2 T allele.	Fig. 1A
**Morphology**	Photography	*Normal*	Fig. 1B
**Characterization of the pluripotent/undifferentiated state**	Marker expression analysis: Qualitative analysis: Immunofluorescence imagingQuantitative analysis: Flowcytometry	SOX2, OCT3/4, NANOG, TRA-1–60, and SSEA-3 were all expressed at levels above 99% in both SBDS-1 and SBDS-1 iso.	Fig. 1C, D
**Karyotypic characterization**	Karyotyping by G-banding	*46XX, Normal, Female*.	Fig. 1E
**Genotyping for the desired genomic alteration/allelic status of the gene of interest**	PCR across the edited site followed by either targeted deep NGS (Next Generation Sequencing) or Sanger sequencing	SBDS1 is heterozygous for the c.258 + 2 T > C mutation, whereas SBDS-1 iso is homozygous for the wild-type c.258 + 2 T allele.	Fig. 1A
	N/A	*N/A*	*N/A* N/A
**Verification of the absence of random plasmid integration events**	N/A	*N/A*	N/A
**Evidence of genetic identity for the parental and the reported genetically-modified cell lines**	STR analysis	*24 sites tested, matched*	*Submitted in the archive with the journal*
**Mutagenesis / genetic modification outcome analysis**	N/A N/A N/A		
**Off-target nuclease activity analysis**	PCR Followed by NGS at top 5 nominated off-target sites.	Off-target assessment was performed using deep targeted sequencing of the top predicted Cas nuclease-based off- target sites to evaluate any unintended base conversions.	Supplementary Fig. 1B
**Specific pathogen-free status**	Mycoplasma	Negative PCR for Mycoplasma	Supplementary Fig. 1A
**Characterization of the differentiation potential**	Directed tri-lineage in-vitro differentiation	*Positive immunostaining for Ectoderm PAX6, Endoderm: FOXA2, and Mesoderm Brachyury (BRACH)*	Fig. 1D
***List of recommended germ layer markers***	As above		
***Overall outcomes of gene editing experiment (OPTIONAL)***	Brief description of the outcomes in terms of clones generated / establishment approach / editing event screening outcomes	*N/A*	N/A
***Donor screening (OPTIONAL)***	HIV 1 + 2 Hepatitis B, Hepatitis C	*N/A*	N/A
***Genotype – additional histocompatibility info (OPTIONAL)***	Blood group genotyping	*N/A*	N/A
	HLA tissue typing	*N/A*	N/A

**Table 2:** Reagents details.

Antibodies and stains used for immunocytochemistry/flow-cytometry			

	Antibody	Dilution	Company Cat # and RRID

*Pluripotency Markers*	*FITC Mouse anti-TRA-1–60*	*1:5*	*BD Biosciences Cat# 560380, BD Biosciences*
	*Alexa Fluor 647 Mouse anti-SSEA-3*	*1:20*	*Cat# 561145, RRID: AB_10893794*
	*BV421 Mouse ant-OCT3/4*	*1:10*	*BD Biosciences Cat# 565644, RRID:AB_2739320*
	*Alexa Fluor 647Mouse anti-SOX2*	*1:20*	*BD Biosciences Cat# 562139, RRID: AB_10897844*
	*Alexa Fluor 647 Mouse anti-NANOG*	*1:5*	*BD Biosciences Cat# 560791, RRID:AB 1937305*
*Differentiation Markers*	*Rabbit anti-PAX6*	*1:100*	*Thermo Fisher Scientific Cat# 42–6600, RRID: AB_2533534*
	*Rabbit anti-BRACH*	*1:200*	*Thermo Fisher Scientific Cat# PA5-114917, RRID: AB_2899553*
	*Rabbit anti-FOXA2*	*1:100*	*MiUipore Cat# 07–633, RRID:AB_390153*
*Secondary Antibodies*	*Goat anti-mouse IgG*	*1:500*	*Thermo Fisher Scientific Cat# A-11005, RRID: AB_2534073*
	*Goat anti-rabbit IgG*	*1:500*	*Thermo Fisher Scientific Cat# A-11008, RRID: AB_143165*
*Nuclear Stain*	*DAPI*	*1 drop per chamber*	*Invitrogen Cat# P36935*
**Site-specific nuclease**		
*Nuclease information*	TadA-based Cytosine Base Editor	CBE6b variant	
*Delivery method*	Electroporation	Lonza 4D-Nucleofector^™^ X-unit nucleofector, Program CB-150, 20ul cartridge	
Selection/enrichment strategy	Next Generation Sequencing	Low-density seeding was used to enable clonal colony formation. Individual colonies were manually picked, expanded, and analyzed by NGS to identify correctly edited lines	
Primers and Oligonucleotides used in this study			
	Target	Forward/Reverse primer (5′–3′)	
*e.g. Episomal Plasmids (qPCR or RT-PCR)*	*NA*	*NA*	
*e.g. Pluripotency Markers (qPCR)*	*NA*	*NA*	
*e.g House-Keeping Genes (qPCR)*	*NA*	*NA*	
*Genotyping*	SBDS Partial amplification followed by Next generation Sequencing (Illumina adaptors in lower case)	Common Forward: ctacacgacgctcttccgatctAGGACTTTCTCTCCTGCCCT Long (Nested) PCR Reverse: GGCAGAGTTTGCAAAAAGAGGG Short PCR Reverse: cagacgtgtgctcttccgatctTTGCTTGGTTAGTCTTTCCTCCA	
*Targeted mutation analysis/sequencing*	SBDS Partial amplification followed by Sanger Sequencing	For: ATCTAGGGGCGGGGGTTGGGRev: GAAGTGACACTGTGCAGTTCAC	
*Potential random integration-detecting PCRs*	NA	NA	
*gRNA oligonucleotide*	Synthetic single guide RNA (sgRNA)- target Cytosine uppercase.	*caggCgggtaacagctgcag*	
*Genomic target sequence(s)*	5′-caggCgggtaacagctgcag**GAT**-3′ Target cytosine is indicated in bold uppercase; PAM sequence is underlined/bolded.	Genomic target sequence GRCh38, chr7: 66,994,210	
*Bioinformatic gRNA on– and – off-target binding prediction tool used*	gRNA was designed manually and evaluated using CRISPOR	Experimental validation by PCR followed by NGS confirmed precise on-target editing, with no detectable bystander mutations or indels in the editing window.	
*Primers for top off-target mutagenesis predicted site sequencing*	*OT1:* intron:PIWIL2	OT1 F: TTCATTCCCCTCCCGTCCAAATACC	
	OT2: intergenic:RP11-211G23.2-RP11-211G23.1	OT1 R:CCAGCCGGGAGAGCAACCAGCCGAA	
	OT3: intron:COL4A5	OT2 F: CCCAGTCAGGCACCTGGGAGTTGGT	
	OT4: intergenic:ME3-CTD-2005H7.1	OT2 R: AGTGTCTGGTGGGATAAGCTGCCGC	
	OT5: intergenic:PPP1R37-EIF5AP3	OT3 F: ACCAAGGCAGCCCCACTCTTTCCAGT	
		OT3 R: ACACTGGCACTGGTGTTGGCTGTTGT	
		OT4 F: TGTTCCCGCTTTCCACGG	
		OT4 R: AGGTTTCCAAACAAGCGAACAT	
		OT5 F: CCTCTCGTCTCACACTCCAGGTCCCG	
		OT5 R: CAGTGCTGGGCGGGAGAGGTGTGG	
*ODNs/plasmids/RNA molecules used as templates for HDR-mediated site-directed mutagenesis.*	*Backbone modifications in utilized ODNS have to be noted using standard nomenclature.*	N/A	

## Data Availability

Data will be made available on request.

## Appendix A. Supplementary data

Supplementary data containing mycoplasma testing and off-target analysis can be found online at https://doi.org/10.1016/j.scr.2026.104045.
