# Supplementary material for: Generation of iPSC and isogenic gene-corrected lines from a patient with Shwachman Diamond syndrome
Source: Stem Cell Res. Author manuscript; Available in PMC 2026 Nov 9. (PMC13746856; doi:10.1016/j.scr.2026.104045)
Supplement: Supplementary file 1 [file NIHMS2199751-supplement-Supplementary_Material.pdf]

A.

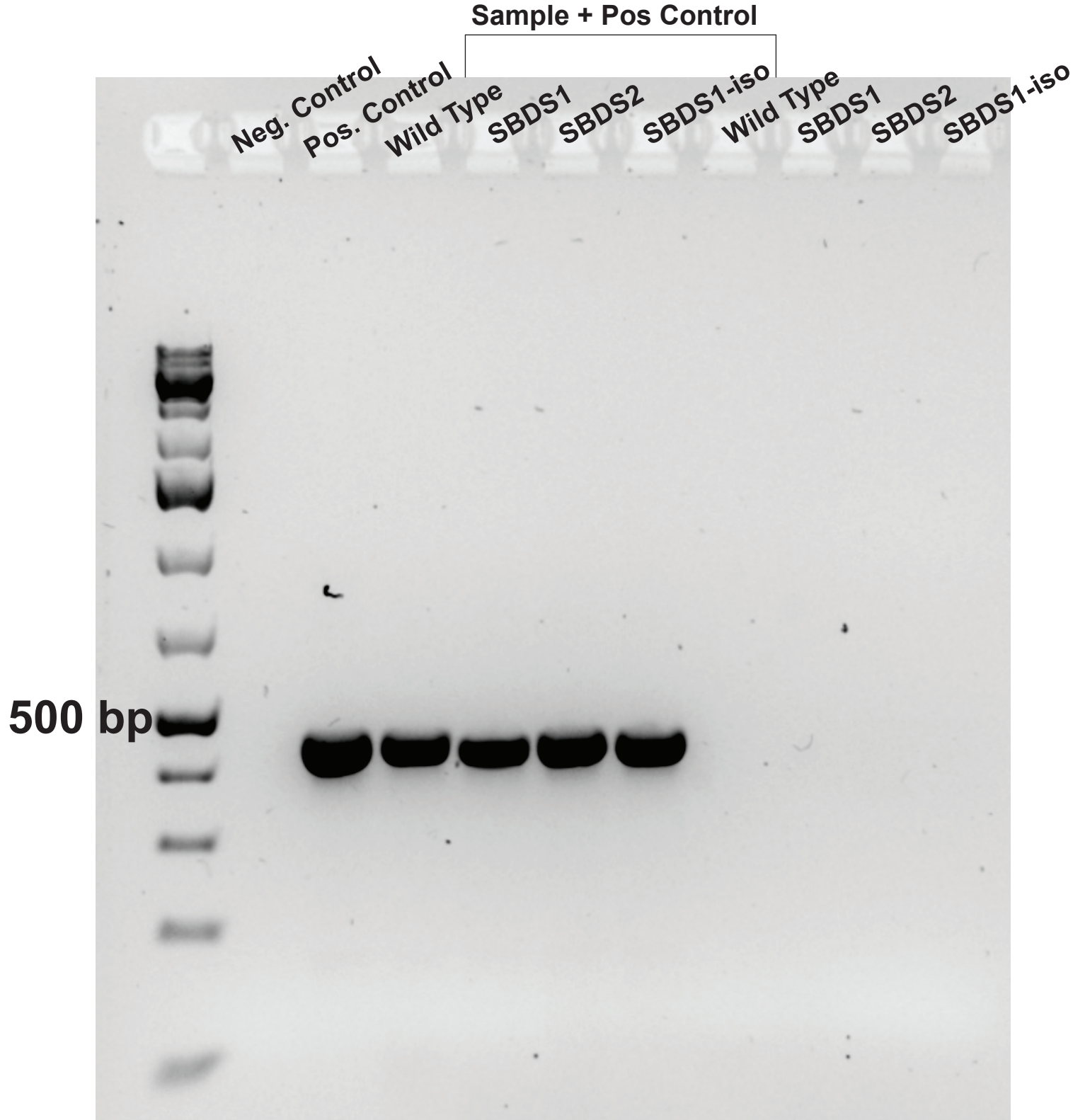

B.

Locus	Reads			
	SBDS1 Unedited	SBDS1 + mRNA Only	SBDS1 edited with AAVS1 Control guide	SBDS1 Edited
intron:PIWIL2	2 (0.1%)	2 (0.1%)	0 (0.0%)	60 (4.0 %)
intergenic:RP11-211G23.2-RP11-211G23.1	1 (0.0%)	0 (0.0%)	0 (0.0%)	1 (0.0%)
intron:COL4A5	1 (0.0%)	0 (0.0%)	1 (0.1%)	1 (0.0%)
intergenic:ME3-CTD-2005H7.1	3 (0.1%)	1 (0.0%)	1 (0.0%)	1 (0.0%)
intergenic:PPP1R37-EIF5AP3	1 (0.0%)	0 (0.0%)	1 (0.1%)	1 (0.1%)

Supplementary Figure 1. Mycoplasma testing and off-target analysis of top 5 predicted off-target sites
